# Digital Decisions: Enhancing Chronic Disease Self-Care Through Digital Health and AI-Enhanced Decision-Making

**DOI:** 10.2196/106648

**Published:** 2026-08-13

**Authors:** Ashley C Griffin, Donna M Zulman

**Affiliations:** 1 Center for Innovation to Implementation, VA Palo Alto Health Care System, 795 Willow Rd, Menlo Park, CA, 94025, United States, 1 6504935000; 2 Center for Biomedical Informatics Research, School of Medicine, Stanford University, Stanford, CA, United States; 3 Division of Primary Care and Population Health, School of Medicine, Stanford University, Stanford, CA, United States

**Keywords:** chronic disease, eHealth, self-care, self-management, AI

## Abstract

Advances in digital health have dramatically changed how patients engage with their health. Rather than relying solely on periodic clinical visits, patients now have access to smartphones, patient portals, wearable devices, and mobile apps that provide support for day-to-day self-care decisions. This commentary discusses the findings of Longhini et al’s systematic review and meta-analysis on the effectiveness of digital health interventions, which found modest improvements in self-care monitoring but limited effects on self-care maintenance and management behaviors. Reflecting on these findings through the lens of dual-process theory, digital health technologies appear to be effective at supporting fast, intuitive processes, such as symptom monitoring, but are less effective at engaging slower, deliberative processes needed for complex decision-making and behavior change. Digital health technologies should evolve from primarily supporting routine self-care activities to enhancing patients’ reflective decision-making processes for sustained behavior change. Emerging AI capabilities offer opportunities to strengthen and bridge these fast and slow cognitive processes by translating complex health information into actionable insights and facilitating patient-clinician communication. Realizing this potential requires careful attention to implementation, including integration into clinical workflows, patient and clinician education, and digital literacy. In addition, digital health teams should adopt standardized implementation frameworks and outcome measures to generate a more robust evidence base. Lastly, human-centered design, patient engagement, and safeguards addressing bias, privacy, and transparency are foundational to ensure that the rapid pace of digital health technology will continue to enhance patient self-care and health outcomes.

## Introduction

Patients are at the forefront of a new era in health care, where care is no longer confined to occasional clinical encounters but embedded into daily life. Throughout the past decade, smartphones, mobile apps, patient portals, and wearable devices have evolved from passive tracking tools into more active platforms that help patients monitor and respond to their own health. Digital health tools can help patients monitor symptoms, identify patterns and trends, and provide guidance for managing their health. These technologies are well positioned to reinforce healthy behaviors through reminders, goal setting, and feedback loops, which are important for sustaining longer-term adherence. Despite their potential to transform chronic disease self-care, evidence for the effectiveness of digital health technologies has been inconclusive.

To address this gap, Longhini et al [1] conducted a systematic review and meta-analysis to examine the impact of digital health interventions on self-care in adults with chronic disease. Across 55 randomized controlled trials including 5889 participants with conditions such as heart failure and diabetes, the researchers observed that interventions were typically multifaceted and included various technologies, behavior change strategies, and involvement from health professionals. For patients with heart failure, interventions demonstrated a modest improvement in self-care monitoring (eg, watching for signs and symptoms, symptom tracking), but showed no clear benefits for self-care maintenance behaviors (eg, diet, physical activity). In diabetes, pooled analyses showed little to no significant improvement in self-care behaviors. There was also no significant overall improvement in medication adherence, although there was variation in results across studies and low certainty of evidence overall. While some individual studies reported positive effects, particularly when interventions incorporated education, clinician feedback, and interactive features, these benefits were not consistently replicated across trials.

## Supporting Self-Care Decision-Making Through Digital Health

The findings from Longhini et al [1] suggest that digital health interventions play an important role in self-care, as evidenced by improvements in monitoring behaviors such as checking for symptoms or monitoring weight. Monitoring changes in signs and symptoms is a critical component of self-care, as changes must be noticed before deciding how to respond. However, *monitoring* represents only one dimension of comprehensive self-care [2]. Other components encompass *maintenance* (eg, dietary behaviors, physical activity, taking medications as prescribed) and *management* (eg, decision-making and taking action in response to symptoms). Maintenance and management require patients to engage in more complex behavioral and cognitive processes, including decision-making, problem-solving, and reflection.

These observations parallel Kahneman’s [3] dual-process theory, which describes how decision-making involves the interaction between fast, intuitive thinking (system 1) and slower, more deliberate reasoning (system 2). Both of these systems are involved in optimal self-care. Routine monitoring and noticing changes can be largely automatic and rely on system 1 processes. In contrast, management tasks such as responding to changes in health and maintenance tasks like adherence to treatment often require more reflection, using system 2. The review by Longhini et al [1] suggests that while digital health interventions may be successful in supporting the faster system 1 processes, they may not be reaching their full potential in engaging the slower system 2 processes required for sustained behavior change. The field of digital health should move beyond supporting the more automatic health behaviors toward actively engaging patients in the reflective decision-making processes involved in self-care.

## Opportunities and Considerations for AI-Enabled Digital Health Tools

**Figure 1.**
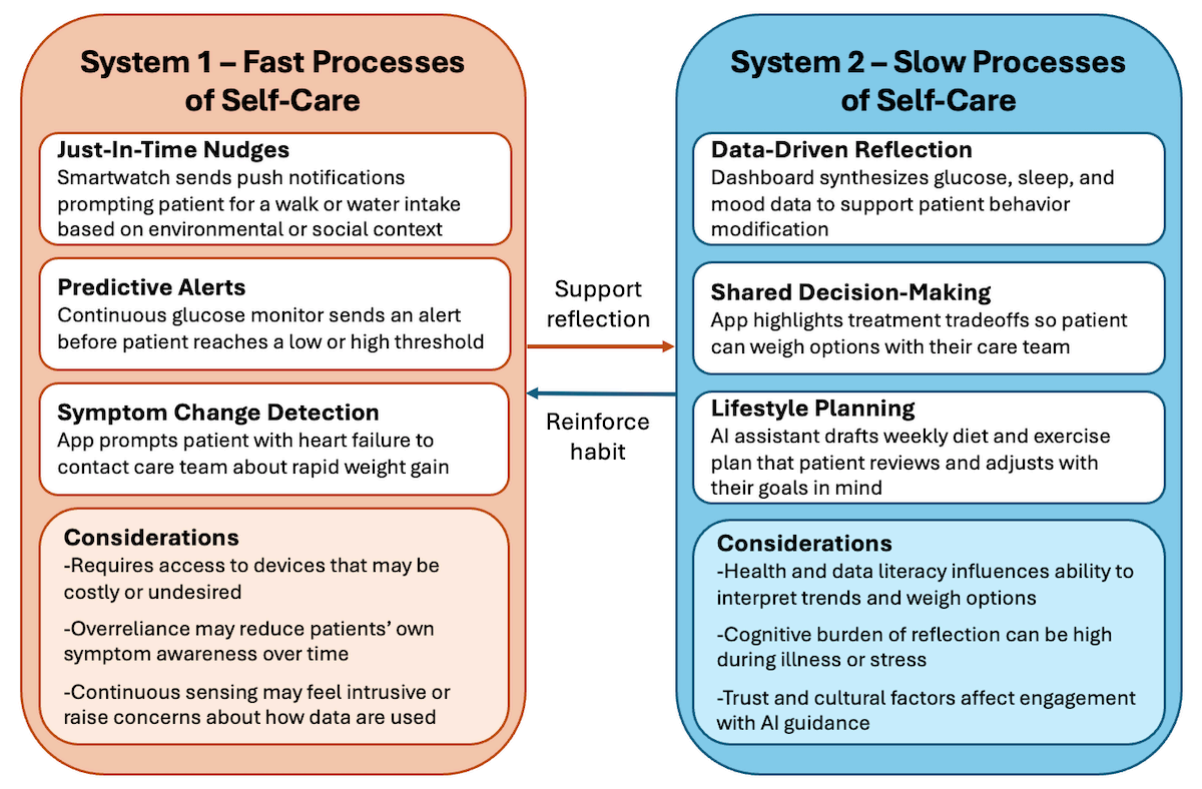
Examples of how AI-enabled digital health tools support self-care cognitive processes.

As digital health interventions increasingly integrate AI, there are opportunities to reinforce and bridge both systems of thinking by translating complex health information into meaningful insights that can be acted upon without extensive cognitive effort (Figure 1). In patients with heart failure, wearables and connected devices that track weight, physical activity, and other physiological measures can help patients recognize patterns associated with worsening symptoms, such as fluid retention [4]. AI could synthesize these signals and highlight changes to facilitate more actionable decision-making. Over time, activities that traditionally require more deliberation, such as determining when to seek medical care, may become more routine and potentially shift from slower processes toward more intuitive decisions. Generative AI tools may further support system 2 processes by helping patients prepare questions for their care team ahead of a visit, plan dietary changes, or develop exercise plans [5,6]. In doing so, these tools could support both immediate action and deeper reflections.

This raises important questions for future work on the ability of AI to influence cognitive processes and support reflection and reasoning. Furthermore, understanding how AI-based tools integrate within the critical role of human supporters, including care teams, caregivers, and peers, has become more important than ever before. Given the current hype surrounding AI, digital health teams should be mindful of algorithmic biases, privacy, transparency, and broader concerns about the negative impact of AI on society [7]. Engaging patients throughout the design and development of AI is paramount to support the creation of human-centered tools [8].

## Challenges of Digital Health Implementation

The promise of newer technologies can only be realized through thoughtful implementation that accounts for an individual’s needs, access, support systems, and environment. In accordance with prior work [9], Longhini et al [1] emphasize the importance of integrating interventions into clinical care using structured implementation strategies, such as providing education for patients and clinicians, embedding tools within workflows, and tailoring interventions based on digital needs and literacy. Recent work has further demonstrated that few digital health studies explicitly apply implementation science frameworks, limiting the ability to understand how contextual factors influence adoption, sustainability, and effectiveness [10]. As technologies are rapidly evolving, digital health teams must adopt standardized frameworks and outcome measures to build a more robust evidence base.

## Conclusion

Digital health interventions show promise for enhancing self-care activities such as symptom monitoring, but opportunities remain to strengthen the more reflective skills required for effective chronic disease management and sustained behavior change. We expect that emerging AI capabilities will help patients navigate the complex decisions involved in maintaining their health, responding to symptoms, and engaging with their care teams. However, achieving this vision requires thoughtful implementation and evaluation strategies that account for the complexity of real-world care and ensure that innovation translates to meaningful improvements.
